# Editorial for the Gut Microbiota Section

**DOI:** 10.3390/microorganisms13061364

**Published:** 2025-06-12

**Authors:** Pramod K. Gopal

**Affiliations:** 1 The New Zealand Institute for Plant and Food Research Limited (PFR), Private Bag 11600, Palmerston North 4442, New Zealand; pramod.gopal@plantandfood.co.nz; 2 Riddet Institute, Massey University, Palmerston North 4410, New Zealand

The gut microbiota—a diverse and dynamic consortium of trillions of microorganisms inhabiting the gastrointestinal tract—plays a pivotal role in host health across species. As the scientific community continues to uncover the profound influence of the gut microbiome on host physiology, we stand at the forefront of a transformative era in biomedical research. From metabolic regulation and immune system modulation to neurobehavioral outcomes, the gut microbiota has emerged as a central determinant of both health and disease.

In this context, we are pleased to invite researchers, clinicians, and interdisciplinary scientists to contribute to the *Gut Microbiota* section of *Microorganisms*, a platform dedicated to advancing the frontiers of microbiome science.

We welcome submissions of original research articles, systematic reviews, meta-analyses, and short communications that investigate:

- Novel microbial mechanisms and pathways relevant to both human and animal health;
- Therapeutic strategies, including next-generation probiotics and prebiotics, as well as faecal microbial transplantation;
- Host–microbiome interactions across physiological systems and species;
- Microbiome-based diagnostics and precision medicine approaches.

Submissions should be grounded in robust methodology and offer clear mechanistic insights or translational relevance. We particularly encourage contributions that incorporate multi-omics technologies, computational modelling, or clinical trial data, as well as studies addressing microbiome diversity across populations and environments.

All manuscripts will undergo a rigorous peer-review process. We emphasise transparency, reproducibility, and adherence to the highest standards of ethical research.

Join us in shaping the future of microbiome science and contributing to a growing body of knowledge that supports microbiota-informed solutions for improved health and well-being.

